# Midwife-Led Home Births in Japan: A 25-Year Retrospective Analysis of Care in Accordance with WHO Recommendations Before and After COVID-19

**DOI:** 10.3390/healthcare14060818

**Published:** 2026-03-23

**Authors:** Mari Murakami, Hiromi Kawasaki, Kimiko Tagawa, Eiko Maehara, Mika Tanaka, Maki Takashima, Kaori Fujita, Satoko Yamasaki, Sae Nakaoka, Mikako Yoshihara, Saori Fujimoto

**Affiliations:** 1 Graduate School of Biomedical and Health Sciences, Hiroshima University, Hiroshima 734-8553, Japan; 2 Department of Nursing, Faculty of Human Health Sciences, Shunan University, Shunan City 745-8566, Japan; 3 Reiko Birth Center, Hiroshima 734-0004, Japan

**Keywords:** home birth, midwifery, COVID-19 pandemic, parturition

## Abstract

**Highlights:**

**What are the main findings?**

- Midwife-led planned home births in Japan aligned with 16 of 25 applicable WHO intrapartum care recommendations, indicating substantial adherence to international standards over 25 years.
- Maternal age, gestational weight gain, and neonatal birth weight shifted significantly following the COVID-19 pandemic.

**What are the implications of the main findings?**

- Independent midwives in Japan provide safe, evidence-based, woman-centered care, supporting physiological birth and positive perinatal outcomes in planned home births.
- Pandemic-related changes in maternal characteristics highlight the need for adaptable community-based perinatal support in Japan’s evolving maternity care system.

**Abstract:**

**Background/Objectives:** In Japan, hospital births predominate, with home births comprising only 0.1% of deliveries. This study assessed how documented practices for planned home births attended by independent midwives align with national guidelines and WHO intrapartum care recommendations, and assess maternal and neonatal differences before and after the COVID-19 pandemic. **Methods:** Records of 430 low-risk pregnant women who received continuous care at a private midwifery home over 25 years were reviewed. After excluding 8 maternal and 22 neonatal transfers, 400 records were analyzed. Descriptive statistics were compared with WHO recommendations and between the pre-pandemic (1999–2019) and post-pandemic (2020–2024) periods. **Results:** All women experienced spontaneous singleton cephalic labors with intermittent fetal heart rate auscultation. The mean gestational age was 277.3 days and the median labor duration was 303.5 min. Labor onset was spontaneous in 83.5% of cases. Nearly half of the women had no perineal lacerations. Postpartum blood loss ≥500 mL occurred in 14.1% of cases. Family presence was nearly universal. Neonates had a mean birth weight of 3129.0 g and high Apgar scores. Skin-to-skin contact occurred in 52.9%; exclusive breastfeeding reached 93.8% at 1 month. Post-pandemic births showed higher maternal age and higher neonatal birth weight, although these differences should be interpreted cautiously due to the small post-pandemic sample. **Conclusions:** Independent midwives provided evidence-based, physiologically oriented care, partially aligning with selected WHO intrapartum recommendations during planned home births. Midwife-led home births may support positive childbirth experiences and favorable maternal/neonatal outcomes for low-risk women. Post-pandemic shifts underscore the need for continued monitoring and flexible, community-based perinatal support, while recognizing the limitations of retrospective, single-site data.

## 1. Introduction

Japan is experiencing a sustained decline in birth rates, accompanied by the consolidation and closure of obstetric and gynecological facilities, intensifying regional disparities in access to maternity care [1,2,3]. These structural changes have contributed to an increase in unassisted childbirths and raised concerns regarding the long-term sustainability of perinatal services [2,4,5]. Although hospital and clinic births now account for nearly all deliveries in Japan [6], this highly medicalized system has prompted questions about whether current practices adequately support women’s autonomy, comfort, and psychosocial well-being [7,8,9].

The COVID-19 pandemic has transformed childbirth environments. Specifically, restrictions on partner attendance, heightened infection concerns, and reduced availability of institutional services led many women to seek alternatives to hospital-based care [6,10,11]. International studies have similarly reported increased interest in home births during the pandemic, reflecting a global reassessment of childbirth settings [10,11]. Research from Europe, North America, and Oceania has shown that pandemic-related policies—such as limiting support persons, reducing prenatal visit frequency, and modifying intrapartum protocols—significantly influenced women’s preferences for out-of-hospital birth [12,13]. In Japan, these developments renewed attention to midwife-led continuity models that emphasize individualized, family-centered care.

Historically, home births have been the norm in Japan, accounting for 95.4% of deliveries in 1950; however, this proportion declined sharply to 0.1% by 1990 as facility-based births became standard [6]. While this transition contributed to the low perinatal and maternal mortality rates in Japan [2,14,15,16,17], emerging evidence highlights potential drawbacks of overmedicalization, including unnecessary interventions, limited mobility during labor, and reduced opportunities for family involvement [7,8,9]. Qualitative studies have suggested that planned home births may enhance women’s autonomy, strengthen relationships with midwives, and promote family cohesion [18]; however, quantitative evidence regarding the content and outcomes of midwife-led home births remains scarce. In response to global concerns about respectful, woman-centered maternity care, the World Health Organization (WHO) issued 56 intrapartum care recommendations in 2018 to promote positive childbirth experiences [19]. These emphasize emotional support, freedom of movement, minimal unnecessary interventions, and effective communication. International implementation studies have shown that adherence to WHO recommendations varies widely across countries, with barriers including institutional protocols, staffing constraints, and inconsistent documentation practices [20]. Midwife-led care—particularly in home birth settings—shares several core features with these principles, and is guided in Japan by the Midwifery Practice Guidelines and Evidence-Based Guidelines for Midwifery Care [21,22]. However, no Japanese studies have yet systematically evaluated how midwife-led home birth practices align with WHO recommendations, or how these practices have evolved, causing a significant evidence gap. Longitudinal, quantitative analyses of midwife-led home births are lacking, and no studies have examined: (1) the extent to which documented practices suggest partial alignment with WHO intrapartum care recommendations, and (2) how maternal and neonatal characteristics differ before and after the COVID-19 pandemic. Given the ongoing restructuring of Japan’s maternity care system and the renewed interest in community-based models, such evidence is needed.

Therefore, this retrospective study aimed to achieve the following: (1) to document practices that suggest partial alignment with WHO intrapartum care recommendations, and (2) to compare maternal and neonatal characteristics before and after the COVID-19 pandemic.

## 2. Materials and Methods

### 2.1. Study Design

This retrospective case series analyzed 25 years of midwifery records to examine the characteristics and outcomes of planned home births attended by independent midwives in Japan. The study evaluated: (1) the alignment of midwifery-led home birth practices with WHO recommendations for intrapartum care supporting positive childbirth experiences [19], and (2) the differences in maternal and neonatal characteristics before and after the COVID-19 pandemic.

### 2.2. Setting

The study was conducted at a private midwifery home in Japan, providing continuous woman-centered care for low-risk pregnant women. The term “private midwifery home” refers to the midwifery practice office where antenatal and postpartum care were provided, not a birth center, and no births took place within the midwifery home facility.

Services included antenatal check-ups, intrapartum support, postpartum care, and newborn assessments. All care adhered to the Midwifery Practice Guidelines [21] and Evidence-Based Guidelines for Midwifery Care [22]. Personal identifiers were removed before analysis to ensure anonymity. All births included in this study occurred in the clients’ private homes.

### 2.3. Sample

**Figure 1:**
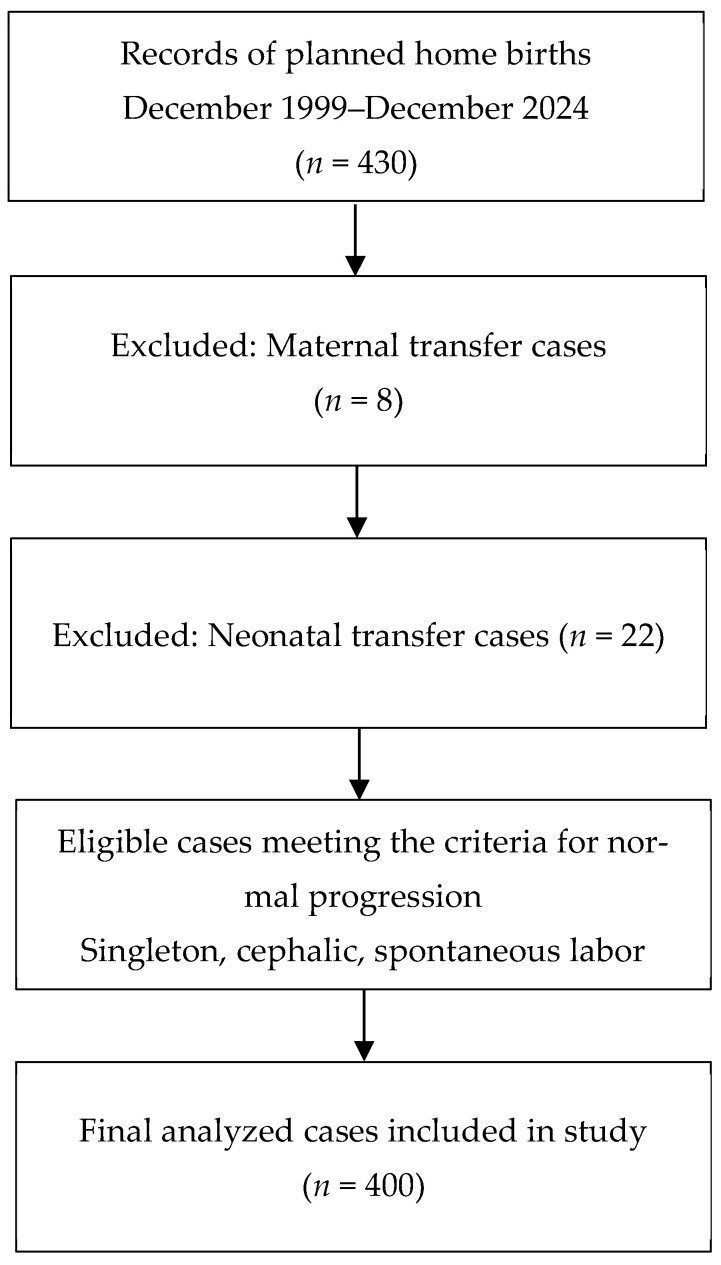
Flowchart of the selection process for planned home birth cases included in the retrospective analysis. A total of 430 records were reviewed; after excluding maternal (*n* = 8) and neonatal (*n* = 22) transfer cases, 400 cases met the criteria for normal cephalic singleton home births and were included in the final analysis.

Among 430 reviewed midwifery records, 400 met the criteria for normal cephalic singleton home births after excluding maternal (*n* = 8) and neonatal (*n* = 22) transfer cases (Figure 1). During the 25-year study period, continued care for each woman was provided by a primary independent midwife. Several other independent midwives supported intrapartum care as secondary midwives, as needed. Therefore, the documented practices in this study reflect the work of a small team of midwives rather than a single practitioner.

The inclusion criteria were as follows:

- Low-risk pregnancy, classified per Japanese midwifery guidelines [21,22]
- Planned home birth
- Cases with continuity of care by the same independent midwife throughout the perinatal period

### 2.4. Data Collection

Data were extracted from the anonymized midwifery records of completed home births. It should be noted that documentation formats and record-keeping procedures evolved over the 25 years; these changes may have influenced data completeness and consistency, particularly for variables such as skin-to-skin contact, breastfeeding initiation, and labor monitoring. This potential documentation bias has been addressed under the ‘Limitations’ section.

Standardized perinatal morbidity indicators, such as neonatal acidosis, NICU admission, or laboratory-based assessments, were not consistently available in this home birth setting and therefore could not be systematically analyzed. Our analysis was consequently limited to variables documented reliably in midwifery records.

Maternal variables included age, height during pregnancy, pre-pregnancy weight, late-pregnancy weight, onset of labor, rupture of membranes, labor duration, fetal heart rate auscultation, perineal outcomes, estimated blood loss, fetal presentation, breastfeeding initiation, and need for transfer. Neonatal variables included gestational age, Apgar scores, birth weight, 1-month weight, and need for transfer.

Because the documentation formats changed throughout the 25-year period, certain variables, such as skin-to-skin contact and breastfeeding initiation, were not consistently recorded. These variations may have introduced documentation bias; this fact was considered when interpreting the results.

### 2.5. Statistical Analysis

Descriptive statistics (means, standard deviations, frequency, and ratio) were used to summarize maternal and neonatal characteristics. However, variables showing substantial variability or skewness, such as labor duration and gestational weight gain, median and interquartile ranges were calculated. Percentages excluded missing data. Analyses were conducted using JMP^®^ Student Edition version 19 (SAS Institute Inc., Cary, NC, USA).

All cases were categorized into two groups according to childbirth timing:

- Pre-pandemic births: 1999–2019
- Post-pandemic births: 2020–2024

After assessing distributional characteristics, Welch’s *t*-test (α = 0.05) was used to examine differences in maternal age, gestational age, and neonatal birth weight. Welch’s test was selected as a more robust alternative to the standard Student’s *t*-test because the assumption of equal variances could not be assured, and the sample sizes between the groups were highly unbalanced. Given the substantial variability and non-parametric distributions observed for gestational weight gain and labor duration, the Wilcoxon rank-sum test (Mann–Whitney U test) was used to compare medians.

We did not perform multivariable regression analyses (e.g., linear or logistic regression) for two main reasons. First, the study followed a descriptive retrospective case series design, and several key covariates required for robust multivariable modeling—such as standardized BMI, detailed socioeconomic indicators, and consistently documented lifestyle factors—were not available across the entire 25-year period. Second, the post-pandemic subgroup (*n* = 35) was too small to support stable multivariable models without a high risk of overfitting. Missing data were treated as missing completely at random and were excluded from the denominator calculations for percentage-based analyses.

### 2.6. Comparison with WHO Recommendations

Two researchers independently reviewed all of the available records, coding each WHO recommendation using predefined operational indicators (e.g., presence of intermittent fetal heart rate auscultation, documentation of maternal mobility, timing of cord clamping); any discrepancies between researchers were resolved through discussion. A detailed list of these indicators is provided in Supplementary Table.

Collected variables were compared with the WHO’s 2018 intrapartum care recommendations to assess alignment with international standards [19]. Among 33 “recommended” or “context-specific” recommendations, 10 were excluded: 2 were deemed commonplace for routine recording (e.g., respectful maternity care, effective communication), 7 were outside the scope of Japanese midwifery homes (e.g., epidural or opioid analgesia, prophylactic uterotonics), and 1 was related to institutional births. Thus, 23 items extractable from midwifery records were analyzed.

For each of the 23 recommendations, we defined the objective operational indicators based on the available documentation (e.g., presence or absence of intermittent fetal heart rate auscultation, documented the timing of cord clamping, or recorded family presence). Compliance was assessed descriptively according to whether these indicators were documented as having occurred. The reproducibility and transparency of the assessment are inherently limited, and should be interpreted with caution as these criteria were derived from retrospective records rather than prospectively standardized protocols.

### 2.7. Ethical Considerations

This study was approved by the Ethical Committee for Epidemiology of Hiroshima University. Informed consent was obtained via an opt-out process via the maternity home’s official website. Data were anonymized before analysis. Hiroshima University’s research team analyzed de-identified data, including admission date and time, delivery date and time, delivery method, and neonatal outcomes.

## 3. Results

### 3.1. Descriptive Statistics of Maternal and Neonatal Characteristics

Among the 400 analyzed cases, the mean maternal age was 32.7 years (standard deviation [SD], 4.1). Spontaneous labor onset occurred in 83.5% of cases, while premature rupture of membranes occurred in 16.5%. The median (IQR) of labor duration was 303.5 (196–473) min, while that for primipara was 535 (356–845) and for multiparous was 270 (184–405) min. Nearly half of the included women (48.6%) had no perineal lacerations, and 42.3% had only first-degree tears. Blood loss of ≥500 mL occurred in 14.1% of patients. In this home birth setting, intrapartum and immediate postpartum blood loss were estimated visually using absorbent materials (e.g., pads, towels) and, when feasible, by weighing blood-soaked materials. The observed rate of blood loss ≥500 mL (14.1%) was within the commonly reported range for vaginal births (approximately 5–15%) in Japanese obstetric practice, and was not associated with severe maternal complications in this cohort. Family presence during labor and birth was nearly universal (99.5%) (Table 1 and Table 2). Where family composition was documented, partners and older children were most commonly present during labor and birth, whereas the presence of grandparents or other relatives was less common. Detailed information on the number and specific relationships of accompanying family members was not consistently available across all records.

The mean neonatal birth weight was 3129.0 g (SD, 338.9); the mean Apgar scores were 9.5 at 1 min and 9.9 at 5 min; skin-to-skin contact lasting <1 h occurred in 52.9% of cases. At 1 month, infants weighed a mean of 4109.2 g (SD = 493.0), and exclusive breastfeeding was achieved in 93.8% (Table 1 and Table 2). However, documentation of the timing and duration of early skin-to-skin contact (SSC) was incomplete, particularly in the earlier period of the 25 years. Because the start time of SSC was commonly missing, some instances of immediate or prolonged contact may not have been captured in the categorical variable used for analysis, and more detailed analyses of skin-to-skin duration or its associations with outcomes were not feasible. 

### 3.2. Alignment with WHO Recommendations for Intrapartum Care

**Figure 2:**
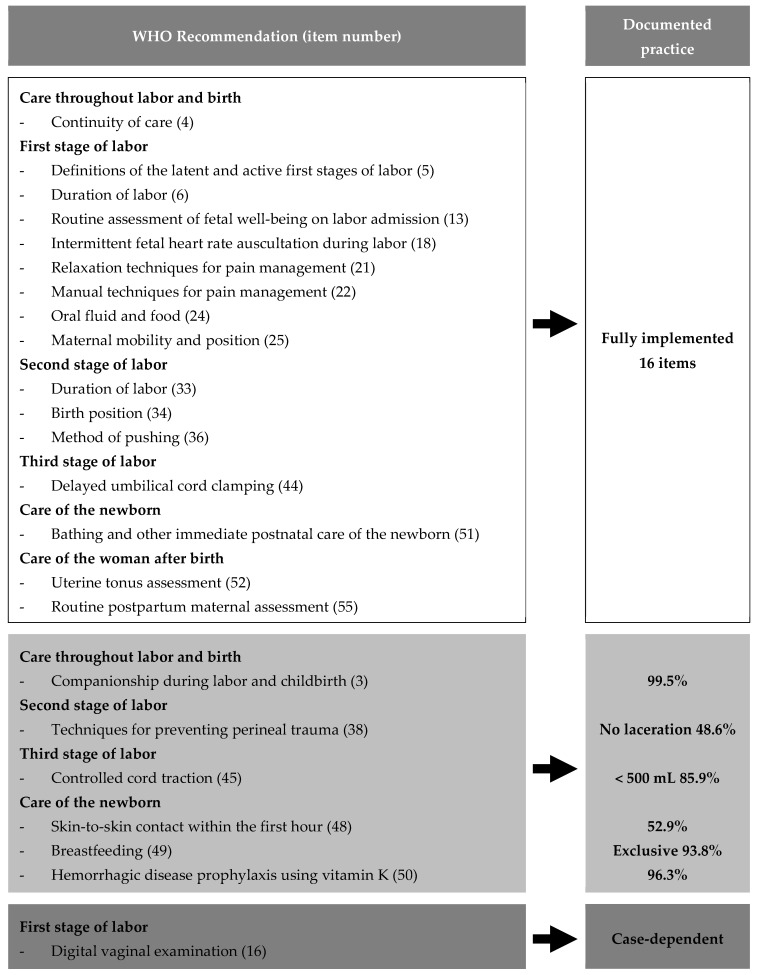
Alignment between WHO intrapartum care recommendations and documented midwifery practices.

Among the 23 applicable WHO intrapartum care recommendations, 16 were implemented. Most implemented items related to supportive care, freedom of movement, non-pharmacological labor support, and delayed cord clamping (Figure 1). Figure 2 illustrates the mapping of the WHO recommendations to documented midwifery practices.

This figure shows the alignment of 23 relevant WHO recommendations for positive childbirth experiences with practices in 400 planned home births attended by independent midwives. Among these, 16 were fully implemented, including the continuity of care, intermittent fetal heart rate monitoring, maternal mobility, and delayed cord clamping. Most implemented recommendations related to supportive care, freedom of movement, and non-intervention practices. Additional indicators, such as family presence (99.5%), exclusive breastfeeding at 1 month (93.8%), and skin-to-skin contact within the first hour (52.9%), adhered to WHO standards. One recommendation (digital vaginal examination) was contingent on specific cases, whereas six were partially implemented or quantified.

### 3.3. Comparison of Maternal and Neonatal Characteristics Before and After the COVID-19 Pandemic

Comparison of maternal and neonatal characteristics pre-/post-pandemic revealed significant differences in maternal age between the pre-pandemic group (M = 32.6, SD = 4.0, *n* = 365) and the post-pandemic group (M = 34.7, SD = 4.6, *n* = 35), t(39.18) = 2.67, *p* = 0.011, with a statistical power of 0.84. Similarly, neonatal birth weight was significantly higher in the post-pandemic group (M = 3270.2, SD = 357.4, *n* = 35) than in the pre-pandemic group (M = 3115.5, SD = 334.4, *n* = 365), t(39.92) = 2.46, *p* = 0.018, with a statistical power of 0.74. In contrast, no statistically significant differences were observed in gestational age (*p* = 0.059). Furthermore, analysis revealed no statistically significant differences in the median gestational weight gain (*p* = 0.81) or the duration of labor (*p* = 0.55).

## 4. Discussion

This study found that independent midwifery care during planned home births in Japan documented practices that partially aligned with selected WHO recommendations for intrapartum care, supporting a positive childbirth experience [19]. High rates of spontaneous labor onset (83.5%), minimal medical interventions (e.g., 100% intermittent fetal heart rate auscultation, no episiotomy), early breastfeeding initiation (93.8% exclusive at 1 month), and low rates of maternal (e.g., 14.1% blood loss ≥500 mL) and neonatal complications reflect a physiological, woman-centered model of care. These findings align with the results of prior studies on the benefits of midwifery-led care in promoting autonomy, satisfaction, and favorable perinatal outcomes [6,7,23].

### 4.1. Key Findings and Implications

Midwifery care at the studied clinic exemplified WHO principles for a positive birth experience, including respect for physiological processes, emotional support, and continuity of care. High rates of exclusive breastfeeding at 1 month (93.8%) and skin-to-skin contact indicate that this care supports early mother-infant bonding, consistent with factors that enhance maternal confidence and reduce postpartum depression risk [24]. These rates align with an integrated, family-centered approach, potentially reflecting its positive impact [25]. Overall, documented practices suggested partial alignment with selected WHO intrapartum care recommendations, rather than full alignment, due to limitations inherent in retrospective record-based data.

Notably, the home birth setting enabled nearly universal family presence during labor and childbirth (99.5%), which was crucially restricted in hospitals during the COVID-19 pandemic. Although COVID-19 was reclassified to a lower-risk category in Japan, many hospitals limit birth attendance to one person, commonly excluding siblings and grandparents. Such restrictions may hinder older siblings’ psychosocial adjustment and reduce family bonding opportunities. In contrast, home birth environments facilitate family involvement, aligning with the WHO emphasis on emotional support and respectful care [24].

In 3.7% of cases, parents declined oral vitamin K administration after informed consent, citing religious beliefs. Japanese guidelines from the Japan Pediatric Society recommend oral vitamin K2 syrup for healthy full-term infants to prevent neonatal bleeding disorders [26]. Future efforts must provide parental health education and secure informed consent to uphold newborns’ best interests, while respecting religious beliefs.

### 4.2. Interpretation of Changes Before and After COVID-19

The pre- and post-pandemic cohorts differed significantly in terms of maternal age and neonatal birth weight. These trends mirror Japan’s rising maternal age [1] and international pandemic-related changes in maternal behaviors and lifestyles [27]. In addition, modest neonatal birth weight increases were identified, possibly due to altered maternal metabolic status or reduced occupational stress during lockdowns [28]. Even among low-risk pregnant women, pandemic-related lifestyle and behavioral changes affected maternal and neonatal outcomes.

Participants in this study received continuous face-to-face, midwife-led care via planned home births during the COVID-19 pandemic, indicating the effective provision of individualized health education and lifestyle guidance throughout pregnancy. This suggests that community-based, midwife-led continuity of care can influence maternal health behaviors and perinatal outcomes during infectious disease outbreaks [29]. For future perinatal support models in local communities, these results indicate that midwife-led home births and community-based midwifery care—emphasizing individualized, woman-centered approaches—offer valuable options during infectious disease periods. Therefore, preserving and transmitting midwifery skills and philosophies adaptable to these changes is essential.

These differences should be interpreted with caution, as they may reflect secular trends, changes in clientele, or documentation variability rather than pandemic effects alone. The small post-pandemic sample size further limits causal interpretation. Accordingly, our interpretations of the potential mechanisms linking pandemic-related lifestyle changes to neonatal birth weight should be regarded as hypotheses generated from existing literature and observational trends, rather than as evidence-based causal conclusions derived from this dataset.

### 4.3. Interpretation in Context

In Japan, most midwives work in hospitals, where institutional protocols and hierarchies may constrain their scope of practice [30,31,32]. Despite low vacancy rates, opportunities for autonomous, continuous care remain limited. This study’s findings highlight the need to preserve and transmit independent midwives’ skills and philosophies—particularly emphasizing physiological birth, emotional support, and family involvement—to hospital settings.

This study reflects broader sociocultural and medical trends in Japan, where medicalized, facility-based births have distanced childbirth from family and home [1,2]. By documenting a midwifery home that integrates traditional woman-centered care with modern practices, it contributes to debates on midwives’ transformative role in future maternity care amid declining birth rates, aging populations, and pandemic disruptions. In this cohort, family presence during labor and birth almost always included the woman’s partner and often older children when documentation was available, illustrating how home birth settings can facilitate a broader family involvement than is typically permitted in institutional environments. This pattern supports the argument that midwife-led continuity models can enhance system resilience and family-centered care during crises, although comparative studies with hospital-based births are needed to substantiate this hypothesis.

The physiological and WHO-consistent practices of midwife-led home births demonstrated here suggest that these models offer a valuable, sustainable, evidence-based option in Japan’s evolving maternity system, potentially improving maternal satisfaction and family-centered values, despite declining birth rates and centralizing services. Prior research indicates midwifery-led continuity models reduce interventions, improve outcomes, and increase satisfaction compared to standard obstetric care [33]. Therefore, expanding access to midwife-led home birth models may contribute to a more resilient and responsive maternity care system, although further evidence is required to confirm this.

### 4.4. Limitations and Future Directions

This study has some limitations. First, it relied on data from a single midwifery home with a relatively small sample size, which restricts the generalizability of the findings. The specific practices, client demographics, and local context at this center may not represent midwife-led home births nationwide in Japan. Although the data are comprehensive for this setting, they do not fully reflect the regional variations in home birth practices or outcomes, thereby limiting the applicability of the observed rates and characteristics. Second, the analysis was based exclusively on midwifery records and lacked qualitative data on women’s subjective birth experiences or postpartum mental health. Some data were missing from these records. Although care aligned with WHO recommendations on intrapartum care for a positive childbirth experience, the absence of women’s perspectives precludes drawing any definitive conclusions about whether these experiences were perceived as positive or contributed to enhanced psychological well-being—a core element of the WHO framework. Third, this study relied exclusively on retrospective midwifery records, which are subject to documentation bias, incomplete data capture, and changes in record-keeping practices over time. Excluding maternal and neonatal transfer cases likely resulted in selection bias, overrepresenting uncomplicated births. Key WHO constructs, such as respectful care, informed choice, and perceived autonomy, could not be assessed from records alone, making conclusions about positive childbirth experience indirect and incomplete. Although multiple independent midwives were involved in providing care during the 25 years, the number of practitioners remained limited. Consequently, the findings may partially reflect the practice style of a small group of midwives, which limits generalizability to all independent midwives in Japan. Fourth, potential selection bias must be considered, as women who choose planned home birth with independent midwives may be more motivated, health-conscious, or better educated than the general obstetric population. Fifth, the retrospective, record-based design introduces potential reporting bias, including the under-documentation of certain practices, such as early skin-to-skin contact or specific family composition during birth. Sixth, long-term neonatal outcomes, including neurodevelopmental and health trajectories beyond the first month, were not available, precluding assessment of longer-term safety. Finally, the absence of standardized perinatal morbidity indicators and the inability to perform multivariable regression analyses mean that residual confounding cannot be excluded, and causal inferences regarding observed associations should be avoided.

To address these limitations, future research should include qualitative interviews with women and their families to assess perceptions of home birth and its effects on maternal satisfaction, mental health, and parenting confidence. Efforts should prioritize training to improve the precision and timeliness of midwifery record documentation, thereby enhancing communication with women and families. Moreover, given the small post-pandemic cohort in the present study, larger comparative analyses of pre- and post-pandemic samples—ideally across diverse care settings—would yield more robust evidence on pandemic-related shifts in home birth preferences, outcomes, and perceptions. Future studies should include multi-site designs, standardized prospective data collection, explicit reporting of provider characteristics and care team composition, and inclusion of transfer cases and comparison groups to improve generalizability.

## 5. Conclusions

This 25-year retrospective analysis of planned home births attended by independent midwives in Japan revealed that documented practices partially aligned with selected WHO intrapartum care recommendations and reflected a physiological, woman-centered model of care. High rates of spontaneous labor onset, minimal medical intervention, early breastfeeding, and strong family involvement illustrate the potential strengths of midwife-led continuity models in supporting positive childbirth experiences for low-risk women. The differences observed between pre- and post-pandemic cohorts in maternal age and neonatal birth weight may reflect broader societal trends and pandemic-related lifestyle changes. However, these findings should be interpreted cautiously, given the small post-pandemic sample and the inherent limitations of retrospective record-based data. Although the results cannot be generalized to all independent midwives or home birth settings in Japan, this study nevertheless provides rare longitudinal evidence on midwife-led home births and highlights the value of community-based, individualized maternity care. As Japan’s maternity system continues to evolve amid declining birth rates and changing service structures, midwife-led continuity models may represent a meaningful option for supporting family-centered, respectful care. Future research should incorporate multi-site designs, prospective standardized data collection, qualitative perspectives from women and families, and inclusion of transfer cases and comparison groups to more fully evaluate the safety, experiences, and system-level contributions of midwife-led home birth care.

## Figures and Tables

**Table 1:** Descriptive statistics for maternal and neonatal characteristics (*n* = 400).

**Item, Unit**	**Average**	**Standard Deviation**
Pregnant age, year	32.7	4.1
Pregnancy height, m	1.6	0.05
Pre-pregnancy weight, kg	51.1	6.2
Late pregnancy weight, kg	60.5	6.9
Gestational period, day	277.3	7.0
Apgar Score, 1 min/5 min	9.5/9.9	0.8/0.3
Neonatal birth weight, g	3129.0	338.9
Infant weight in the first month, g	4109.2	493.0
**Item, unit**	**Median**	**IQR**
Length of labor, minute	303.5	196–473

**Table 2:** Frequency distribution of maternal and neonatal characteristics (*n* = 400).

Item	Frequency	Percentage
** ** **Primipara or multiparous**		
Primipara	69	(17.3)
Multiparous	331	(82.7)
** ** **Start of delivery**		
Onset of labor pains	334	(83.5)
Premature rupture of the membranes	66	(16.5)
** ** **Fetal heart rate auscultation**		
Intermittent	400	(100.0)
** ** **Amount of intrapartum bleeding**		
<500 mL	340	(85.9) *
≥500 mL	56	(14.1) *
Missing (No record)	4	-
** ** **Perineal laceration**		
No	193	(48.6) *
1st degree	168	(42.3) *
2nd degree	36	(9.0) *
Missing (No record)	3	-
** ** **Companion during labor and childbirth**		
Family and midwives	398	(99.5)
Only midwives	2	(0.5)
** ** **Skin-to-skin contact: SSC**		
SSC contact with mothers <1 h	184	(52.9) *
SSC contact with mothers after 1 h	162	(46.6) *
No	2	(0.6) *
Missing (No record)	52	-
** ** **Breastfeeding rate in the first month**		
Exclusive breastfeeding	375	(93.8)
Breastfeeding and formula	25	(6.2)
** ** **Hemorrhagic disease prophylaxis using vitamin K**		
1 mg of vitamin K administered orally	385	(96.3)
No (Parents refused with informed consent due to religious wishes)	15	(3.7)

* Percentages were calculated after excluding missing data.

## Data Availability

The data are not publicly available due to privacy and ethical restrictions.

## Supplementary Materials

The following supporting information can be downloaded at: https://www.mdpi.com/article/10.3390/healthcare14060818/s1, Supplementary Table. WHO recommendations Intrapartum care for a positive childbirth experience.
