# Supplementary material for: Midwife-Led Home Births in Japan: A 25-Year Retrospective Analysis of Care in Accordance with WHO Recommendations Before and After COVID-19
Source: Healthcare (Basel). 2026 Mar 23;14(6):818. doi: 10.3390/healthcare14060818 (PMC13026313; doi:10.3390/healthcare14060818)
Supplement: Supplementary file 1 [file healthcare-14-00818-s001.zip › healthcare-4159778-supplementary.pdf]

Supplementary Table. WHO recommendations Intrapartum care for a positive childbirth experience

Summary list of recommendations on intrapartum care for a positive childbirth experience

Care option	Recommendation	Category of recommendation
**Care throughout labour and birth**		
Respectful maternity care	1. Respectful maternity care – which refers to care organized for and provided to all women in a manner that maintains their dignity, privacy and confidentiality, ensures freedom from harm and mistreatment, and enables informed choice and continuous support during labour and childbirth – is recommended.	Recommended
Effective communication	2. Effective communication between maternity care providers and women in labour, using simple and culturally acceptable methods, is recommended.	Recommended
Companionship during labour and childbirth	3. A companion of choice is recommended for all women throughout labour and childbirth.	Recommended
Continuity of care	4. Midwife-led continuity-of-care models, in which a known midwife or small group of known midwives supports a woman throughout the antenatal, intrapartum and postnatal continuum, are recommended for pregnant women in settings with well functioning midwifery programmes.[a]	Context-specific recommendation
**First stage of labour**		
Definitions of the latent and active first stages of labour	5. The use of the following definitions of the latent and active first stages of labour is recommended for practice.<br>— The latent first stage is a period of time characterized by painful uterine contractions and variable changes of the cervix, including some degree of effacement and slower progression of dilatation up to 5 cm for first and subsequent labours.<br>— The active first stage is a period of time characterized by regular painful uterine contractions, a substantial degree of cervical effacement and more rapid cervical dilatation from 5 cm until full dilatation for first and subsequent labours.	Recommended
Duration of the first stage of labour	6. Women should be informed that a standard duration of the latent first stage has not been established and can vary widely from one woman to another. However, the duration of active first stage (from 5 cm until full cervical dilatation) usually does not extend beyond 12 hours in first labours, and usually does not extend beyond 10 hours in subsequent labours.	Recommended
Progress of the first stage of labour	7. For pregnant women with spontaneous labour onset, the cervical dilatation rate threshold of 1 cm/hour during active first stage (as depicted by the partograph alert line) is inaccurate to identify women at risk of adverse birth outcomes and is therefore not recommended for this purpose.	Not recommended
	8. A minimum cervical dilatation rate of 1 cm/hour throughout active first stage is unrealistically fast for some women and is therefore not recommended for identification of normal labour progression. A slower than 1-cm/hour cervical dilatation rate alone should not be a routine indication for obstetric intervention.	Not recommended
	9. Labour may not naturally accelerate until a cervical dilatation threshold of 5 cm is reached. Therefore the use of medical interventions to accelerate labour and birth (such as oxytocin augmentation or caesarean section) before this threshold is not recommended, provided fetal and maternal conditions are reassuring.	Not recommended

[a] Integrated from *WHO recommendations on antenatal care for a positive pregnancy experience*.

Care option	Recommendation	Category of recommendation
Labour ward admission policy	10. For healthy pregnant women presenting in spontaneous labour, a policy of delaying labour ward admission until active first stage is recommended only in the context of rigorous research.	Research-context recommendation
Clinical pelvimetry on admission	11. Routine clinical pelvimetry on admission in labour is not recommended for healthy pregnant women.	Not recommended
Routine assessment of fetal well-being on labour admission	12. Routine cardiotocography is not recommended for the assessment of fetal well-being on labour admission in healthy pregnant women presenting in spontaneous labour.	Not recommended
	13. Auscultation using a Doppler ultrasound device or Pinard fetal stethoscope is recommended for the assessment of fetal well-being on labour admission.	Recommended
Perineal/pubic shaving	14. Routine perineal/pubic shaving prior to giving vaginal birth is not recommended.[a]	Not recommended
Enema on admission	15. Administration of enema for reducing the use of labour augmentation is not recommended.[b]	Not recommended
Digital vaginal examination	16. Digital vaginal examination at intervals of four hours is recommended for routine assessment of active first stage of labour in low-risk women.[a]	Recommended
Continuous cardiotocography during labour	17. Continuous cardiotocography is not recommended for assessment of fetal well-being in healthy pregnant women undergoing spontaneous labour.	Not recommended
Intermittent fetal heart rate auscultation during labour	18. Intermittent auscultation of the fetal heart rate with either a Doppler ultrasound device or Pinard fetal stethoscope is recommended for healthy pregnant women in labour.	Recommended
Epidural analgesia for pain relief	19. Epidural analgesia is recommended for healthy pregnant women requesting pain relief during labour, depending on a woman's preferences.	Recommended
Opioid analgesia for pain relief	20. Parenteral opioids, such as fentanyl, diamorphine and pethidine, are recommended options for healthy pregnant women requesting pain relief during labour, depending on a woman's preferences.	Recommended
Relaxation techniques for pain management	21. Relaxation techniques, including progressive muscle relaxation, breathing, music, mindfulness and other techniques, are recommended for healthy pregnant women requesting pain relief during labour, depending on a woman's preferences.	Recommended
Manual techniques for pain management	22. Manual techniques, such as massage or application of warm packs, are recommended for healthy pregnant women requesting pain relief during labour, depending on a woman's preferences.	Recommended
Pain relief for preventing labour delay	23. Pain relief for preventing delay and reducing the use of augmentation in labour is not recommended.[b]	Not recommended
Oral fluid and food	24. For women at low risk, oral fluid and food intake during labour is recommended.[b]	Recommended
Maternal mobility and position	25. Encouraging the adoption of mobility and an upright position during labour in women at low risk is recommended.[b]	Recommended
Vaginal cleansing	26. Routine vaginal cleansing with chlorhexidine during labour for the purpose of preventing infectious morbidities is not recommended.[a]	Not recommended
Active management of labour	27. A package of care for active management of labour for prevention of delay in labour is not recommended.[b]	Not recommended

[a] Integrated from *WHO recommendations for prevention and treatment of maternal peripartum infections.*
[b] Integrated from *WHO recommendations for augmentation of labour.*

Care option	Recommendation	Category of recommendation
Routine amniotomy	28. The use of amniotomy alone for prevention of delay in labour is not recommended.*	Not recommended
Early amniotomy and oxytocin	29. The use of early amniotomy with early oxytocin augmentation for prevention of delay in labour is not recommended.*	Not recommended
Oxytocin for women with epidural analgesia	30. The use of oxytocin for prevention of delay in labour in women receiving epidural analgesia is not recommended.*	Not recommended
Antispasmodic agents	31. The use of antispasmodic agents for prevention of delay in labour is not recommended.*	Not recommended
Intravenous fluids for preventing labour delay	32. The use of intravenous fluids with the aim of shortening the duration of labour is not recommended.*	Not recommended
**Second stage of labour**		
Definition and duration of the second stage of labour	33. The use of the following definition and duration of the second stage of labour is recommended for practice.<br>— The second stage is the period of time between full cervical dilatation and birth of the baby, during which the woman has an involuntary urge to bear down, as a result of expulsive uterine contractions.<br>— Women should be informed that the duration of the second stage varies from one woman to another. In first labours, birth is usually completed within 3 hours whereas in subsequent labours, birth is usually completed within 2 hours.	Recommended
Birth position (for women without epidural analgesia)	34. For women without epidural analgesia, encouraging the adoption of a birth position of the individual woman's choice, including upright positions, is recommended.	Recommended
Birth position (for women with epidural analgesia)	35. For women with epidural analgesia, encouraging the adoption of a birth position of the individual woman's choice, including upright positions, is recommended.	Recommended
Method of pushing	36. Women in the expulsive phase of the second stage of labour should be encouraged and supported to follow their own urge to push.	Recommended
Method of pushing (for women with epidural analgesia)	37. For women with epidural analgesia in the second stage of labour, delaying pushing for one to two hours after full dilatation or until the woman regains the sensory urge to bear down is recommended in the context where resources are available for longer stay in second stage and perinatal hypoxia can be adequately assessed and managed.	Context-specific recommendation
Techniques for preventing perineal trauma	38. For women in the second stage of labour, techniques to reduce perineal trauma and facilitate spontaneous birth (including perineal massage, warm compresses and a "hands on" guarding of the perineum) are recommended, based on a woman's preferences and available options.	Recommended
Episiotomy policy	39. Routine or liberal use of episiotomy is not recommended for women undergoing spontaneous vaginal birth.	Not recommended
Fundal pressure	40. Application of manual fundal pressure to facilitate childbirth during the second stage of labour is not recommended.	Not recommended

* Integrated from *WHO recommendations for augmentation of labour.*

Care option	Recommendation	Category of recommendation
**Third stage of labour**		
Prophylactic uterotonics	41. The use of uterotonics for the prevention of postpartum haemorrhage (PPH) during the third stage of labour is recommended for all births.[a]	Recommended
	42. Oxytocin (10 IU, IM/IV) is the recommended uterotonic drug for the prevention of postpartum haemorrhage (PPH).[a]	Recommended
	43. In settings where oxytocin is unavailable, the use of other injectable uterotonics (if appropriate, ergometrine/ methylergometrine, or the fixed drug combination of oxytocin and ergometrine) or oral misoprostol (600 µg) is recommended.[a]	Recommended
Delayed umbilical cord clamping	44. Delayed umbilical cord clamping (not earlier than 1 minute after birth) is recommended for improved maternal and infant health and nutrition outcomes.[b]	Recommended
Controlled cord traction (CCT)	45. In settings where skilled birth attendants are available, controlled cord traction (CCT) is recommended for vaginal births if the care provider and the parturient woman regard a small reduction in blood loss and a small reduction in the duration of the third stage of labour as important.[a]	Recommended
Uterine massage	46. Sustained uterine massage is not recommended as an intervention to prevent postpartum haemorrhage (PPH) in women who have received prophylactic oxytocin.[a]	Not recommended
**Care of the newborn**		
Routine nasal or oral suction	47. In neonates born through clear amniotic fluid who start breathing on their own after birth, suctioning of the mouth and nose should not be performed.[c]	Not recommended
Skin-to-skin contact	48. Newborns without complications should be kept in skin-to-skin contact (SSC) with their mothers during the first hour after birth to prevent hypothermia and promote breastfeeding.[d]	Recommended
Breastfeeding	49. All newborns, including low-birth-weight (LBW) babies who are able to breastfeed, should be put to the breast as soon as possible after birth when they are clinically stable, and the mother and baby are ready.[e]	Recommended
Haemorrhagic disease prophylaxis using vitamin K	50. All newborns should be given 1 mg of vitamin K intramuscularly after birth (i.e. after the first hour by which the infant should be in skin-to-skin contact with the mother and breastfeeding should be initiated).[d]	Recommended
Bathing and other immediate postnatal care of the newborn	51. Bathing should be delayed until 24 hours after birth. If this is not possible due to cultural reasons, bathing should be delayed for at least six hours. Appropriate clothing of the baby for ambient temperature is recommended. This means one to two layers of clothes more than adults, and use of hats/caps. The mother and baby should not be separated and should stay in the same room 24 hours a day.[f]	Recommended

[a] Integrated from *WHO recommendations for the prevention and treatment of postpartum haemorrhage*.
[b] Integrated from the *WHO Guideline: delayed cord clamping for improved maternal and infant health and nutrition outcomes*.
[c] Integrated from *WHO Guidelines on basic newborn resuscitation*.
[d] Integrated from *WHO Recommendations for management of common childhood conditions: evidence for technical update of pocket book recommendations*.
[e] Integrated from *WHO recommendations on newborn health*.
[f] Integrated from *WHO recommendations on postnatal care of the mother and newborn*.

Care option	Recommendation	Category of recommendation
**Care of the woman after birth**		
Uterine tonus assessment	52. Postpartum abdominal uterine tonus assessment for early identification of uterine atony is recommended for all women.[a]	Recommended
Antibiotics for uncomplicated vaginal birth	53. Routine antibiotic prophylaxis is not recommended for women with uncomplicated vaginal birth.[b]	Not recommended
Routine antibiotic prophylaxis for episiotomy	54. Routine antibiotic prophylaxis is not recommended for women with episiotomy.[b]	Not recommended
Routine postpartum maternal assessment	55. All postpartum women should have regular assessment of vaginal bleeding, uterine contraction, fundal height, temperature and heart rate (pulse) routinely during the first 24 hours starting from the first hour after birth. Blood pressure should be measured shortly after birth. If normal, the second blood pressure measurement should be taken within six hours. Urine void should be documented within six hours.[c]	Recommended
Postnatal discharge following uncomplicated vaginal birth	56. After an uncomplicated vaginal birth in a health care facility, healthy mothers and newborns should receive care in the facility for at least 24 hours after birth.[c,d]	Recommended

[a] Integrated from *WHO recommendations for the prevention and treatment of postpartum haemorrhage*.
[b] Integrated from *WHO recommendations for prevention and treatment of maternal peripartum infections*.
[c] Integrated from *WHO recommendations on postnatal care of the mother and newborn*.
[d] For the newborn, this includes an immediate assessment at birth, a full clinical examination around one hour after birth and before discharge.

This table highlights in red the 23 items recommended by the WHO[19)] that correspond to entries in the midwifery records used in this study, and serves as a supplement to Figure 2.
